# Identification of endoplasmic reticulum stress-associated genes and subtypes for prediction of Alzheimer’s disease based on interpretable machine learning

**DOI:** 10.3389/fphar.2022.975774

**Published:** 2022-08-19

**Authors:** Yongxing Lai, Xueyan Lin, Chunjin Lin, Xing Lin, Zhihan Chen, Li Zhang

**Affiliations:** ^1^ Department of Geriatric Medicine, Shengli Clinical Medical College of Fujian Medical University, Fujian Provincial Hospital, Fuzhou, China; ^2^ Fujian Provincial Center for Geriatrics, Fujian Provincial Hospital, Fuzhou, China; ^3^ Department of Gastroenterology, Shengli Clinical Medical College of Fujian Medical University, Fujian Provincial Hospital, Fuzhou, China; ^4^ Department of Rheumatology and Immunology, Shengli Clinical Medical College of Fujian Medical University, Fujian Provincial Hospital, Fuzhou, China; ^5^ Department of Nephrology, Shengli Clinical Medical College of Fujian Medical University, Fujian Provincial Hospital, Fuzhou, China

**Keywords:** Alzheimer’s disease, ER stress, machine learning, molecular subtypes, prediction model

## Abstract

**Introduction:** Alzheimer’s disease (AD) is a severe dementia with clinical and pathological heterogeneity. Our study was aim to explore the roles of endoplasmic reticulum (ER) stress-related genes in AD patients based on interpretable machine learning.

**Methods:** Microarray datasets were obtained from the Gene Expression Omnibus (GEO) database. We performed nine machine learning algorithms including AdaBoost, Logistic Regression, Light Gradient Boosting (LightGBM), Decision Tree (DT), eXtreme Gradient Boosting (XGBoost), Random Forest, K-nearest neighbors (KNN), Naïve Bayes, and support vector machines (SVM) to screen ER stress-related feature genes and estimate their efficiency of these genes for early diagnosis of AD. ROC curves were performed to evaluate model performance. Shapley additive explanation (SHAP) was applied for interpreting the results of these models. AD patients were classified using a consensus clustering algorithm. Immune infiltration and functional enrichment analysis were performed via CIBERSORT and GSVA, respectively. CMap analysis was utilized to identify subtype-specific small-molecule compounds.

**Results:** Higher levels of immune infiltration were found in AD individuals and were markedly linked to deregulated ER stress-related genes. The SVM model exhibited the highest AUC (0.879), accuracy (0.808), recall (0.773), and precision (0.809). Six characteristic genes (RNF5, UBAC2, DNAJC10, RNF103, DDX3X, and NGLY1) were determined, which enable to precisely predict AD progression. The SHAP plots illustrated how a feature gene influence the output of the SVM prediction model. Patients with AD could obtain clinical benefits from the feature gene-based nomogram. Two ER stress-related subtypes were defined in AD, subtype2 exhibited elevated immune infiltration levels and immune score, as well as higher expression of immune checkpoint. We finally identified several subtype-specific small-molecule compounds.

**Conclusion:** Our study provides new insights into the role of ER stress in AD heterogeneity and the development of novel targets for individualized treatment in patients with AD.

## Introduction

Alzheimer’s disease (AD) is a severe progressive neurodegenerative brain disorder evidenced by amyloid-beta (Abeta) plaques aggregation, tauopathy, and cognitive impairment (Masters and Beyreuther, 1998). It has been reported that approximately 2–8% people suffer from dementia over the past decades, affecting more than 50 million people worldwide (Skolnik and Heung, 2018). Alternations in memory are usually the early clinical manifestation of AD, and as the disease progresses it can progressively impair other cognitive domains, manifesting as the dysfunction of speech and motor, and diminished spatial and motor skills (McKhann et al., 2011). Due to the insidious onset of AD, most patients have missed the optimal treatment stage at the time of first diagnosis. In addition, the inconsistency of clinical symptoms and the distinct pathogenesis make the current targeted drugs for AD unsatisfactory (Nandigam, 2008; Rahimi and Kovacs, 2014). Therefore, thoroughly understanding of the potential mechanisms underlying the AD heterogeneity and identifying novel biomarkers to guide therapeutic strategies against clinical progression of AD is imperative.

Endoplasmic reticulum (ER) is a critical organelle in maintaining the homeostasis of intracellular environment. The disruption of ER homeostasis can lead to ER stress under persistent stress insult, which evidenced by the alternations in cellular Ca^+^ concentration and the over-accumulation of unfolded or misfolded proteins (Adamopoulos et al., 2014; Leprivier et al., 2015), eventually resulting in various protein-folding diseases, including AD (Salminen et al., 2009; Li et al., 2015). Relevant researches demonstrated that AD patients and mouse exhibited persistent changes in ER stress pathways including the enhanced phosphorylation of PERK and its downstream molecular eIF2alpha, as well as the increased activity of ATF4 and CHOP, which are positively correlation with the severity of AD (Braak scores) (Marwarha et al., 2017). In addition, other studies proved that ER stress-induced CHOP activation may participate in triggering AD-like pathology via promoting oxidative damage and reinforcing Abeta production (McCullough et al., 2001; Ghribi et al., 2006). These studies suggested that ER stress might be a promising target for the treatment of AD. However, the specific biological mechanisms of ER in regulating AD progression has not been fully elucidated and require further exploration.

In the present study, we comprehensively explored the expression profiles of ER stress-related genes between normal and AD subjects and the correlation between differentially expressed genes (DEGs) associated with ER stress immune characteristics. Subsequently, we compared the performance of nine machine learning algorithms including AdaBoost, Logistic Regression, Light Gradient Boosting (LightGBM), Decision Tree (DT), eXtreme Gradient Boosting (XGBoost), Random Forest, K-nearest neighbors (KNN), Naïve Bayes, and support vector machines (SVM) and determine six characteristic genes among 17 differentially expressed ER stress-related genes, which enable to precisely predict AD progression. Moreover, we proposed novel molecular subtypes in AD patients associated with ER and predicted distinct subtype-specific small-molecule compounds, which may provide a theoretical basis for developing effective therapeutic strategies for AD prevention and treatment.

## Materials

### Data acquisition and pre-processing

Raw gene expression profiles of AD patients were obtained from GSE33000, GSE5281, GSE122063, and GSE97760 microarray datasets of the Gene Expression Omnibus (GEO) website database using the R package of “GEOquery”. In the GSE33000 dataset, there are 157 healthy individuals and 310 AD brain tissue samples, detected by Rosetta/Merck Human 44k 1.1 microarray. The GSE5281 datasets contains 74 normal subjects and 87 AD brain tissue samples, analyzed by Affymetrix Human Genome U133 Plus 2.0 Array. The GSE122063 dataset comprises 44 normal and 56 AD brain tissue samples, detected by Agilent-039494 SurePrint G3 Human GE v2 8 × 60K Microarray 039381. The GSE97760 includes 10 control and nine AD peripheral blood specimens, analyzed by Agilent-039494 SurePrint G3 Human GE v2 8 × 60K Microarray 039381. Subsequently, to obtain a sufficient number of samples for further analysis, we combined the gene expression profiles of three datasets (GSE33000, GSE5281, and GSE122063) including 275 normal and 453 AD samples based on the Combat algorithm of “sva” R package (Leek et al., 2012). The GSE97760 was selected as external validation set.

### Identification of ER stress-associated DEGs

The ER stress-related genes were accessed from two gene sets (GOBP response to endoplasmic reticulum stress and GOBP regulation of response to endoplasmic reticulum stress) of Molecular Signature Database v7.0 (MSigDB). The “limma” R package (Ritchie et al., 2015) was utilized to perform gene analysis of inter-sample differences, and ER stress-related DEGs were determined in consistent with the criteria of | log2 (fold change) |>0.5 and adjusted *p* value <0.001.

### Functional enrichment analysis

Gene Ontology (GO) analysis including biological processes (BP), molecular functions (MF), and cellular components (CC) was conducted to evaluate the biological functions of ER stress-related genes using the R package of “ClusterProfiler” (Yu et al., 2012). The remarkable enriched functions were determined based on the adjusted *p* value less than <0.05. The differences of enriched functions and pathways between distinct ER stress-related subtypes were assessed using GSVA enrichment analysis based on the “GSVA” R package (Hänzelmann et al., 2013). Briefly, Two gene sets (“c2. cp.kegg.v7.4. symbols” and “c5. go.bp.v7.5.1. symbols”) downloaded from MSigDB database were utilized as the input files for the subsequent GSVA analysis. Differential enrichment functions and pathways were identified by calculating the GSVA scores between distinct ER stress-related subtypes using the R package of “limma”. The |t value of GSVA score| greater than two were determined as remarkably enriched functions and pathways.

### Analysis of immune cell infiltrations

The CIBERSORT algorithm with an LM22 gene feature matrix was utilized to evaluate immune cell subtypes in each sample on the basis of the gene expression profiles. The *p*-value for the inverse fold product of each sample were calculated based on the Monte Carlo sampling. The differences in immune cell abundances between distinct groups were estimated using Wilcoxon rank sum testing and a value of *p* less than 0.05 was considered statistically different. The correlation between differential immune cells with ER stress-related genes was analyzed using Spearson correlation analysis.

### Construction and explanation of machine learning

On the basis of expression profiles of differentially expressed ER stress-related genes, we applied the Python package “PyCaret” for establishing nine distinct machine learning models including AdaBoost, Logistic Regression, Light Gradient Boosting (LightGBM), Decision Tree (DT), eXtreme Gradient Boosting (XGBoost), Random Forest, K-nearest neighbors (KNN), Naïve Bayes, and support vector machines (SVM). The classification of diseases was regarded as response variable, and the ER stress-related DEGs were considered as explanatory variables. A total of 728 samples (275 normal and 453 AD) were randomly divided into training (70%) and validation (30%) set. The LR model is a popular classification model enable to accurately predict the expectation of the binary dependent variable based on the regression coefficients (Fitzmaurice and Laird, 2001). The Decision Tree model is a tree-like model including a hierarchy of decision nodes/signature thresholds, and the structures of trees are vulnerable to data distribution and complexity (Speybroeck, 2012). AdaBoost, LightGBM, and XGBoost models are the optimized distributed gradient boosting libraries possess excellent predictive performance via transforming a set of weak variables to strong variables (Freund et al., 1999; Chen and Guestrin, 2016; Ke et al., 2017). The Random Forest model is a multiple classification trees that combines various decision trees through majority voting, eventually exhibiting high tolerance for outliers and noise (Ishwaran, 2015). The KNN model is a non-parametric algorithm based on computing the distance between all samples, classifying observations by assigning them to the class of their nearest k neighboring samples. The Naïve Bayes model is a classifier on the basis of the Bayes theorem for predicting conditional probabilities. The SVM model, an algorithm constructing a linear-decision surface over the features, thus being able to distinctly classify data points (Meyer and Wien, 2001). Each algorithm performs a grid search of hyper-parameters based on 10-fold cross-validated in the training dataset to explore the best set of hyper-parameters.

To estimate the performance of multiple machine learning models, we calculated and compared the areas under the precision-recall (PR) curve and ROC curve (AUC). In addition, to comprehensively compare their performance, we also reported true positive (TP) true negative (TN), false positive (FP), false negative (FN), accuracy, F1 score, Kappa, MCC. Moreover, we conducted the Shapley Additive exPlanation (SHAP) values to provide global and local interpretation of each feature within machine learning models based on the Python package of “SHAP” (Lundberg and Lee, 2017). SHAP values could exhibit how feature variable contributes positively or negatively to the prediction of the outcome.

### Construction of a nomogram

A total of six characteristic ER stress-related genes were incorporated to construct a nomogram based on the R package of “rms”. The calibration curve was applied for estimating the accuracy of the nomogram and the clinical significance of the nomogram was evaluated using the decision curve analysis.

### External validation analysis

The GSE97760 dataset was applied for external validating the ability of six characteristic ER stress-related genes (Supplementary Table S2) to distinguish AD from non-AD normal, and the AUC curves were plotted using the R package of “pROC”.

### Unsupervised clustering of AD patients

Initially, a total of six characteristic ER stress-related genes were obtained according to previously reported (Tsvetkov et al., 2022). We applied the unsupervised clustering analysis (“ConsensusClusterPlus” R package) (Wilkerson and Hayes, 2010) for classifying the 453 AD samples into different clusters by using the k-means algorithm with 1,000 iterations. We chose a maximum subtype number k (*k* = 6) and the optimal subtype number was comprehensively evaluated base on the cumulative distribution function (CDF) curve, consensus matrix, and consistent cluster score (>0.9). t-Distributed Stochastic Neighbor Embedding (tSNE) analysis was performed to demonstrate the distribution difference between ER stress subtypes and was visualized via the package of “ggplot2”.

### Connectivity map and mechanism of action analysis

The Connectivity Map database (CMap, https://clue.io/) was utilized to explore candidate small-molecule compounds targeting the subtype1-specific and subtype2-specific DEGs. Briefly, the top 150 most upregulated and downregulated subtype-specific genes were considered as the input genes to inquire the CMap database. The top 60 potential compounds were selected for MoA analysis on the basis of the compounds enrichment scores.

### Statistical analysis

All statistical testings were performed using R software (version 4.1.0) Wilcoxon sum-rank testing or Student’s t-testing was applied for analyzing the difference between the two groups. The correlation analysis among various variables was conducted using Spearman’s correlation testing. All statistical *p*-values calculated were two-sided, and a value of *p* < 0.05 was considered as statistical significance.

## Results

### Dysregulation of ER stress regulators and alterations in the immunity in AD patients

**FIGURE 1:**
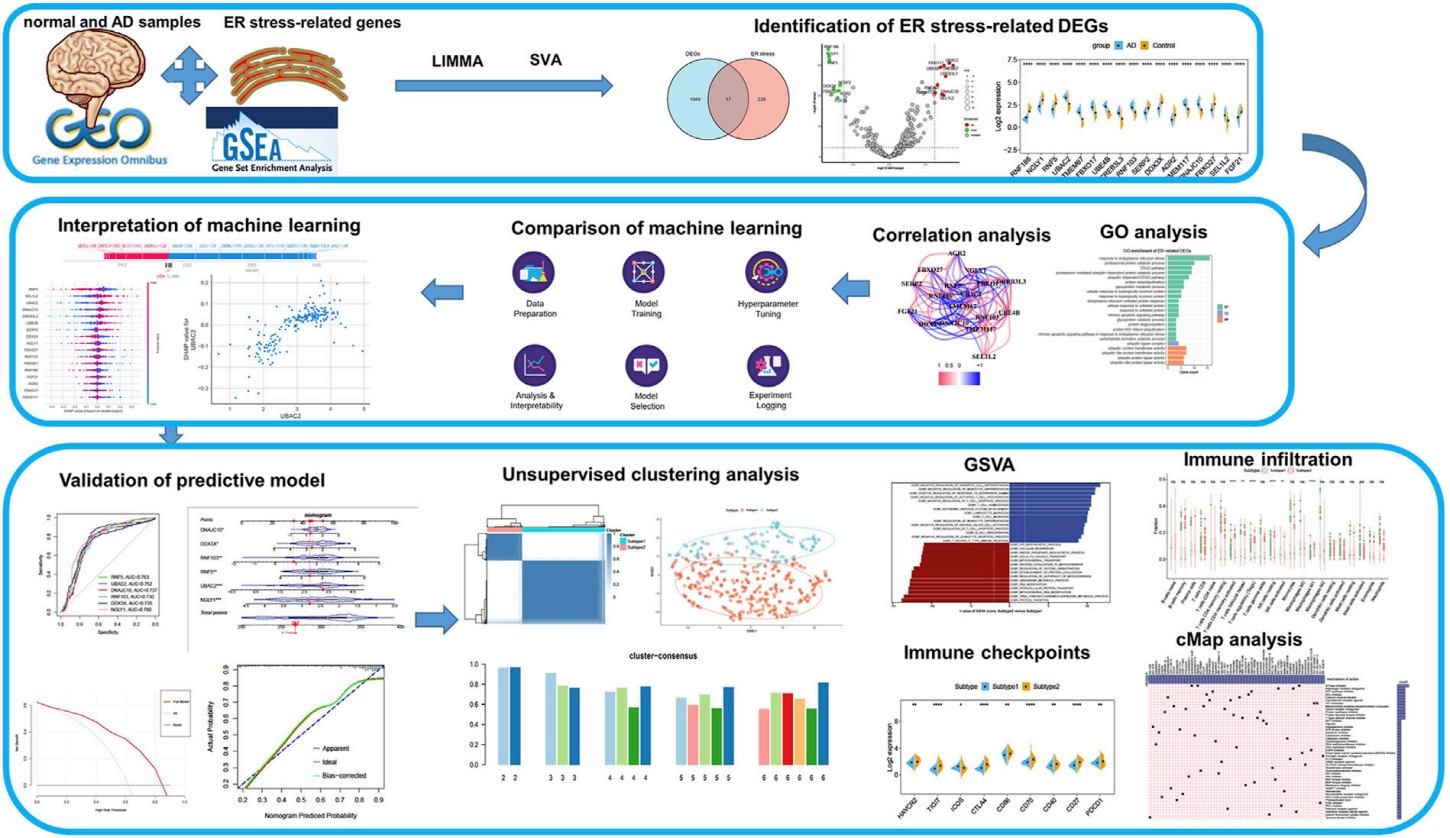
The study flow chart.

**FIGURE 2:**
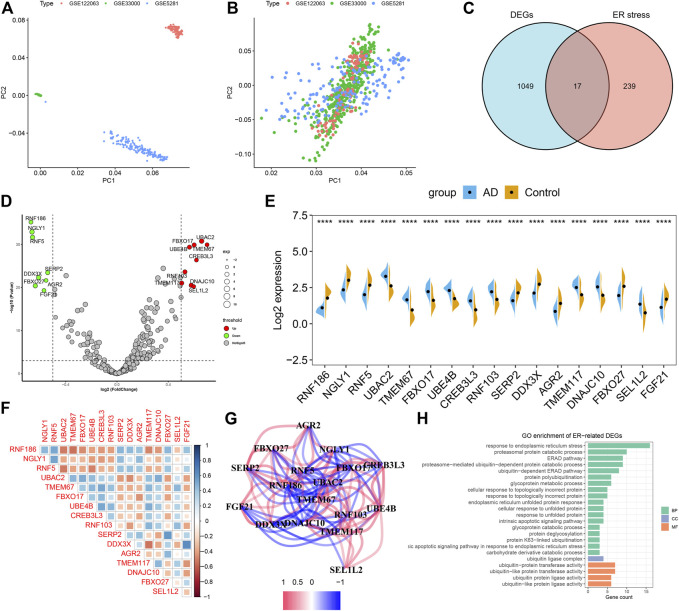
Identification of dysregulated CRGs in AD. **(A,B)** Principal component analysis (PCA) of three datasets before **(A)** and after **(B)** batch effect removal. **(C)** Representative venn diagram shows 17 ER stress-related DEGs. **(D)** Representative volcano plot shows the upregulated and downregulated DEGs associated with ER stress. **(E)** Representative violin diagram showed the expression of 17 ER stress-related DEGs between AD and non-AD controls. ****p < 0.0001. **(F)** Correlation analysis among 17 ER stress-related DEGs. Blue and red color represents positive and negative correlation, respectively. The area of the rectangular box represents the different correlation coefficients. **(G)** Gene relationship network diagram of 17 ER stress-related DEGs. **(H)** Go enrichment analysis of 17 ER stress-related DEGs.

To investigate the biological functions of ER stress-related genes in the progression of AD, we first combined the expression landscapes of 275 normal and 453 AD brain tissue specimens from the GSE33000, GSE5281, and GSE122063 datasets. A detailed flow chart of our study procedure was presented in Figure 1. Brain tissues from distinct platforms showed remarkably different clustering patterns before batch effect removal (Figure 2A). While grouped into together after batch correlation (Figure 2B). A total of 1066 AD-related DEGs (441 up-regulation and 625 down-regulation) were identified using the DEG method. We next intersected the 256 ER stress-related genes with 1066 AD-related DEGs, and seventeen of which were finally determined as the ER stress-related DEGs in AD patients (Figure 2C, Supplementary Table S1). Among them, the gene expression levels of UBAC2, FBXO17, UBE4B, TMEM67, CREB3L3, RNF103, TMEM117, DNAJC10, and SEL1L2 were markedly greater, RNF186, NGLY1, AGR2, RNF5, SERP2, DDX3X, FBXO27, and FGF21 expression levels were significantly lower in AD patients than that in non-AD normal individuals (Figures 2D,E). Subsequently, these 17 ER stress-related DEGs were correlated to estimate whether ER stress played a critical role in the progression of AD. We found some ER stress regulators such as DD3X and FGF21, exhibited a high degree of synergistic effect (coefficient = 0.63). Meanwhile, RNF5 and UBAC2 demonstrated a significant antagonistic action (coefficient = -0.51). Moreover, the correlation patterns of other ER stress-related genes such as NGLY1 and RNF2, RNF186 AND TMEM67, DD3X and TMEM117, SERP2, and FBXO27 was also meaningful (Figure 2F). The network diagram of gene relationship further clarified the closeness of association among these ER stress-related DEGs (Figure 2G). In addition, the results of functional analysis indicated that these ER stress-related DEGs were primary enriched in classical pathways, such as metabolic process, response to ER stress and unfolded protein, and ubiquitin-related functions (Figure 2H).

**FIGURE 3:**
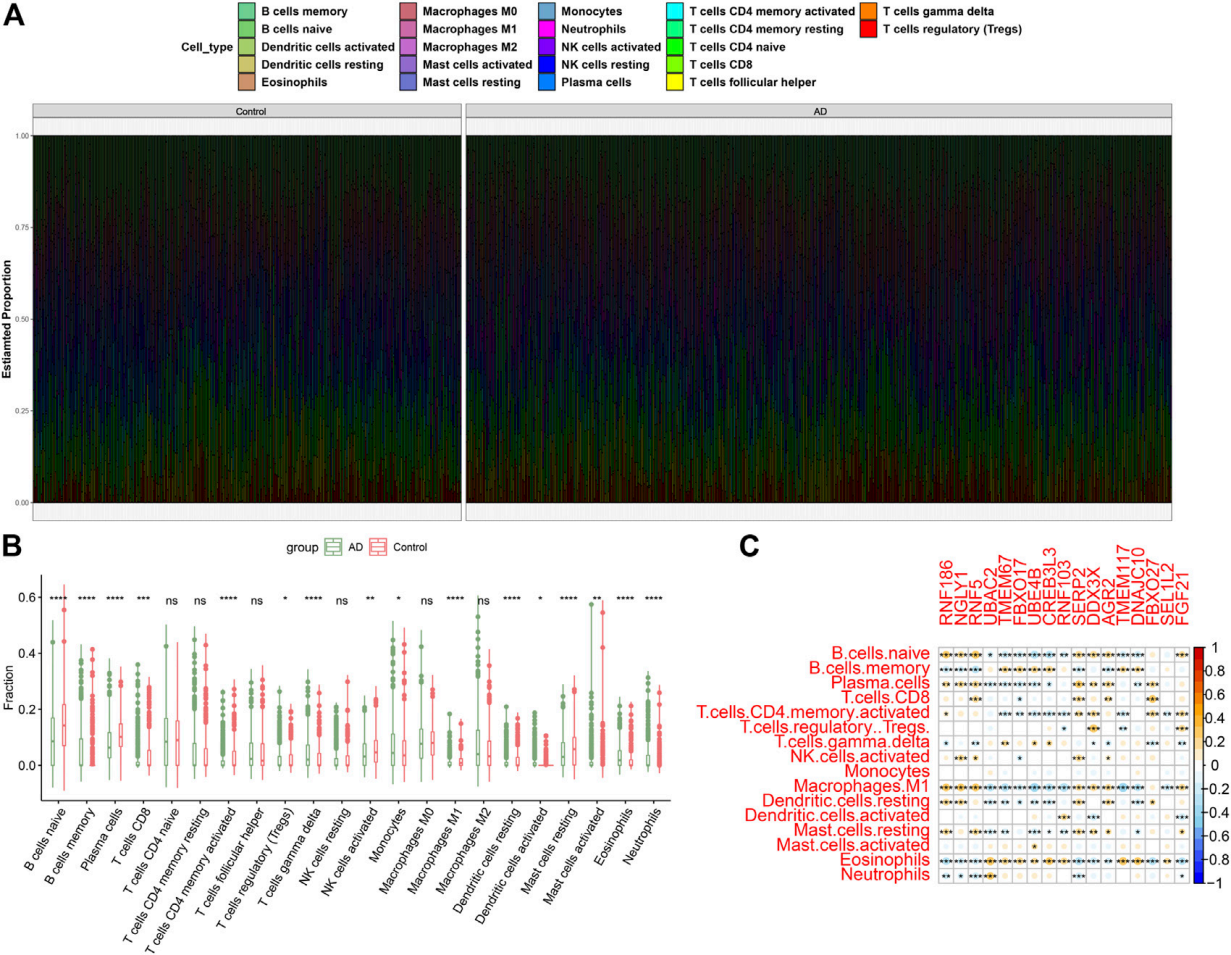
Identification of immune characteristics between AD and non-AD controls. **(A)** The relative proportions of 22 immune cells types between AD and normal individuals. **(B)** Representative boxplots show the differences of infiltrated immune cells between AD and normal individuals. *p < 0.05, **p < 0.01, ***p < 0.001, ****p < 0.0001, ns, no significance. **(C)** Correlation analysis between 17 ER stress-related DEGs and infiltrated immune cells.

To illustrate whether patients with AD existed the altered immune system activity, we conducted immune infiltration analysis and found significant differences in the abundances of 22 immune cell subtypes (Figure 3A). Among them, the infiltration levels of CD8^+^ T cells, regulatory T cells (Tregs), gamma delta T cells, Monocytes, M1 macrophages, resting dendritic cells, activated dendritic cells, activated mast cells, eosinophils and neutrophils were markedly higher in AD patients (Figure 3B), indicating the altered activity of immune system might be involved in the onset and progression of AD. Additionally, correlation analysis results suggested that ER stress-related DEGs were extraordinarily correlated with naïve B cells, memory B cells, plasma cells, activated memory CD4^+^ T cells, M1 macrophages, resting dendritic cells, and eosinophils (Figure 3C), revealing that ER stress-mediated immune cells activation might be the major pathological mechanism causing AD progression.

### Construction and evaluation of machine learning models

**FIGURE 4:**
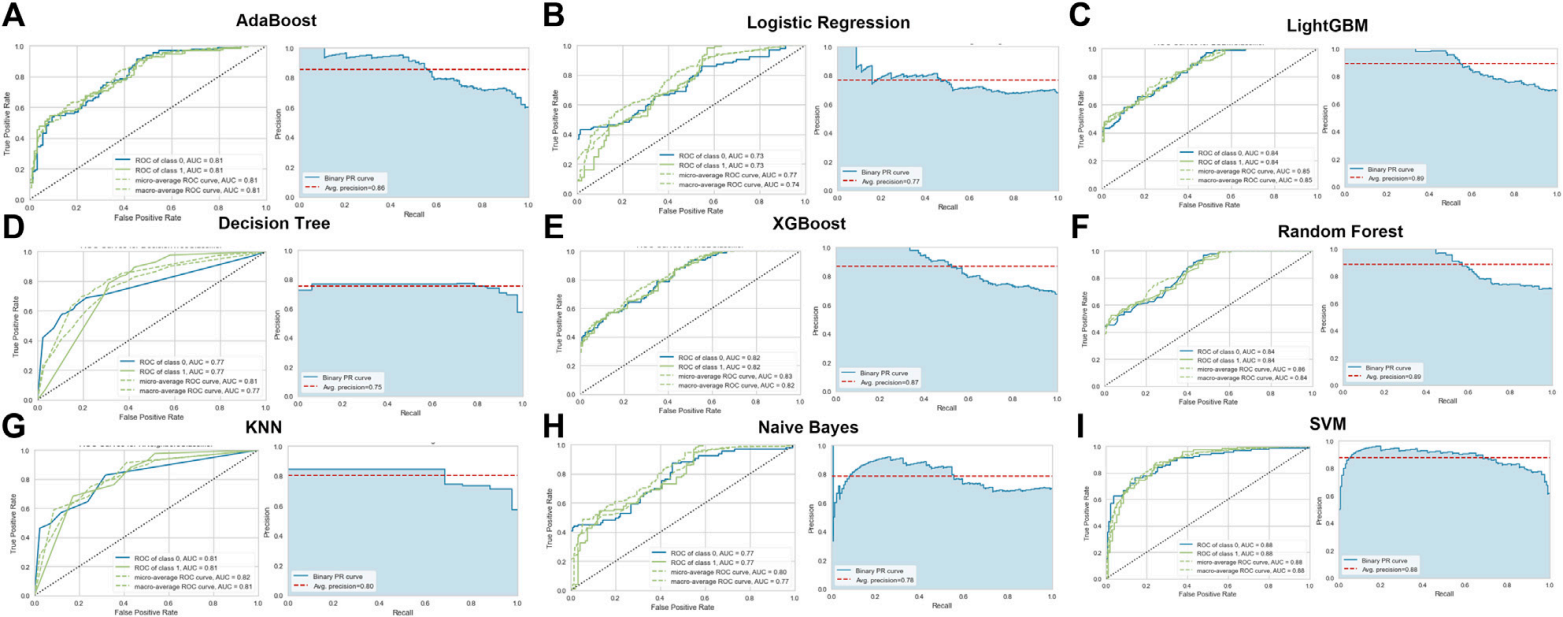
Evaluation of the nine machine learning algorithms based on the area under the ROC curve (AUC) and PR curve. (A) AdaBoost machine learning algorithm. (B) Logistic Regression machine learning algorithm. (C) LightGBM machine learning algorithm. (D) Decision Tree machine learning algorithm. (E) XGBoost machine learning algorithm. (F) Random Forest machine learning algorithm. (G) KNN machine learning algorithm. (H) Naïve Bayes machine learning algorithm. (I) SVM machine learning algorithm.

To identify the optimal machine learning model for diagnosing AD, we acquired the expression profiles of these 17 ER stress-related DEGs and fit them into nine machine learning algorithms. A total of 728 samples (275 normal and 453 AD) were randomly into training sets (*N* = 509) and testinging sets (*N* = 219). Subsequently, we estimated the ability of these machine learning models to discriminate between AD and normal subjects in the testinging sets (Table 1). The SVM model exhibited the highest AUC (0.879) and relative high P-R curve area (0.88), demonstrating the high performance in differentiating between AD samples and normal controls (TP = 115, TN = 61). In addition, the SVM model also showed the best accuracy (0.808), recall (0.773), and precision (0.809). However, The LR model had the lowest AUC (0.733) and accuracy (0.662) (Figure 4; Table 1).

**TABLE 1:** Comparison of the diagnostic efficacy of nine distinct machine learning models.

Model	TP	TN	FP	FN	Accuracy	AUC	Recall	Precision	F1	Kappa	MCC
AdaBoost	100	55	38	26	0.708	0.807	0.794	0.725	0.758	0.392	0.394
Logistic Regression	101	44	49	25	0.662	0.733	0.802	0.673	0.732	0.284	0.292
LightGBM	106	56	37	20	0.740	0.845	0.841	0.741	0.788	0.454	0.460
Decision Tree	105	60	33	21	0.749	0.770	0.833	0.755	0.792	0.477	0.480
XGBoost	108	53	40	18	0.735	0.820	0.857	0.730	0.788	0.441	0.451
Random Forest	114	49	44	12	0.744	0.841	0.905	0.817	0.803	0.452	0.476
KNN	111	53	40	15	0.749	0.808	0.881	0.735	0.801	0.467	0.482
SVM	115	61	32	11	0.808	0.879	0.873	0.809	0.840	0.602	0.605
Naive Bayes	109	43	50	17	0.694	0.768	0.865	0.686	0.764	0.343	0.363

### Global and local explanation of feature genes

**FIGURE 5:**
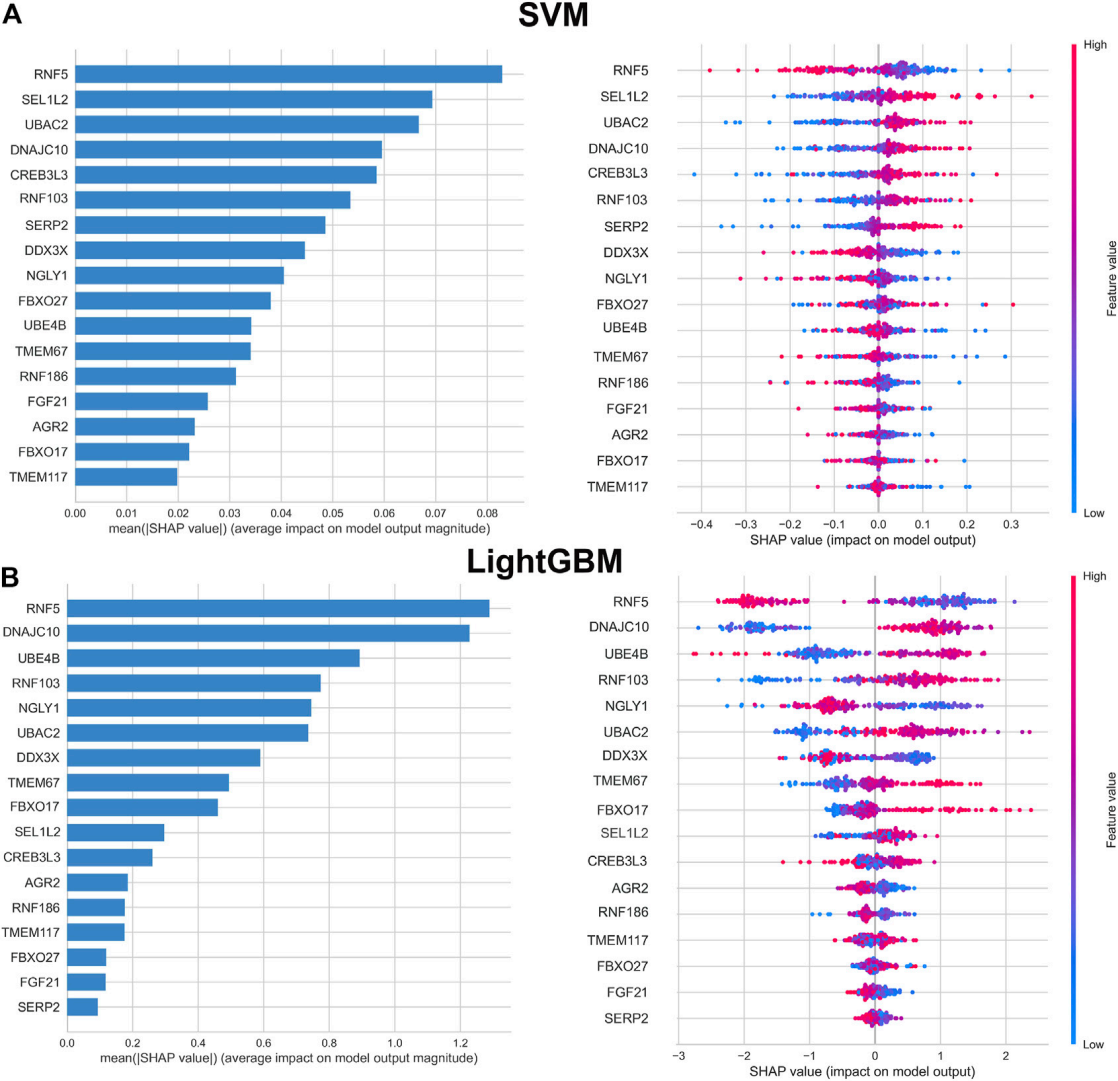
Importance matrix and SHAP summary plot of SVM **(A)** and LightGBM **(B)** machine learning algorithms. Importance matrix exhibit the importance of each variable for predicting AD progression. The SHAP value of each variable is positively correlated with the probability of developing AD. Each dot corresponds to the SHAP value of each sample, and red color corresponds to higher feature values, blue color corresponds to lower feature values.

To further interpret the results of machine learning model, we applied SHAP for computing the influence of each feature to the prediction model. The importance matrix plot for the SVM model indicated that the genes with mean (|SHAP value|) value more than 0.04 were RNF5, SEL1L2, UBAC2, DNAJC10, RNF103, CREB3L3, SERP2, DDX3X, and NGLY1, demonstrating that the top nine features contributed more to the SVM model than other features. In addition, the top nine features in the LightGBM model were as follows: RNF5, DNAJC10, UBE4B, RNF103, NGLY1, UBAC2, RNF186, DDX3X, GBXO17. However, the importance matrix plots could not tell us whether, for example, different expression levels of RNF5 contributed positively or negatively to the probability of AD. Therefore, we depicted the SHAP summary plots for the SVM and LightGBM models, which presented the relationship between the expression levels of features and the degree of high and low SHAP values in the testing dataset. On the SHAP summary plot, feature variables with lower SHAP values were closely associated with an increased risk of developing AD. For example, In the SVM model, enhanced expression of RNF5 corresponded to negative SHAP values and was negatively associated with the prediction of AD occurrence. Contrast that with low expression level of RNF5 was linked to positive SHAP values and exerted a higher marginal influence to the prediction probability of AD. Conversely, the feature SEL1L2 showed the opposite impact. Higher expression level of SEL1L2 had a positive marginal effect on prediction the occurrence of AD, whereas lower expression level was correlated with a negative marginal effect on the prediction of AD occurrence. Other feature variables follow the similar patterns (Figure 5).

### Applying the prediction model

**FIGURE 6:**
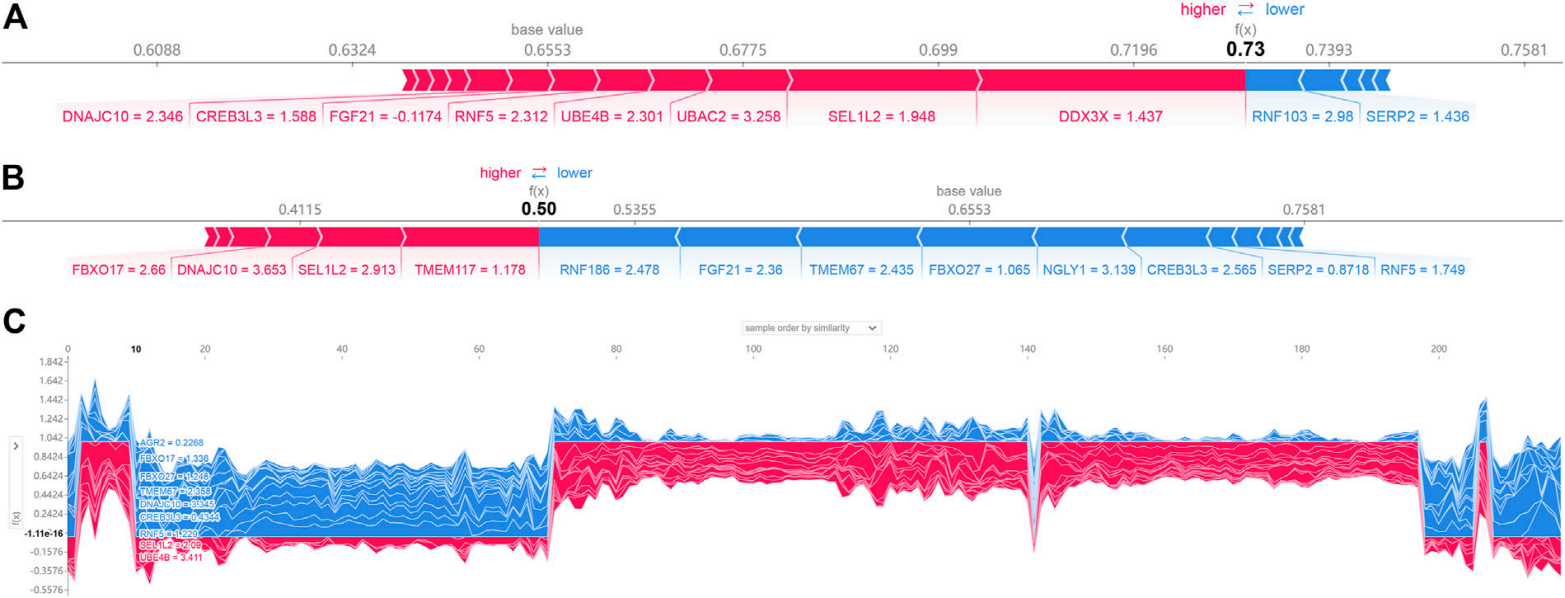
SHAP force plot for a patient **(A)** and normal **(B)** individual; **(C)** SHAP values (global interpretation) for the testing set. The abscissa corresponds to each sample (normal or patient), and the ordinate corresponds to the SHAP value. More red reveals a greater overall risk for developing AD.

SHAP force plots could illustrate profiles of patient and normal subjects. The bold-faced number corresponded to the probability prediction (f(x)), and the base value represented the value predicted without any model input. The blue bar to the right represented a prediction of normal, while the pink bar to the left represented a prediction of increased probability of AD. The length of the colored bars would be facilitated to visualize the extent of the impact on the prediction. The longer the bar, the greater the impact. Figure 6A showed the force plot for a patient individual, which is predicted by the expression of ER stress DEGs. The expression of RNF188, FGF21, TMEM67, FBXO27, NGLY1, CREB3L3 were regarded as the major influential factors concerning AD progression. Figure 6B exhibited the profile of a normal subject. The model considered the expression of DDX3X, SEL1L2, UBAC2, UBEB4 as the apparent influential factors reducing this risk of AD occurrence. Figure 6C depicted the global interpretation for all the samples (including normal and patient subjects) in the testinging set.

### Selection and validation of characteristic genes

**FIGURE 7:**
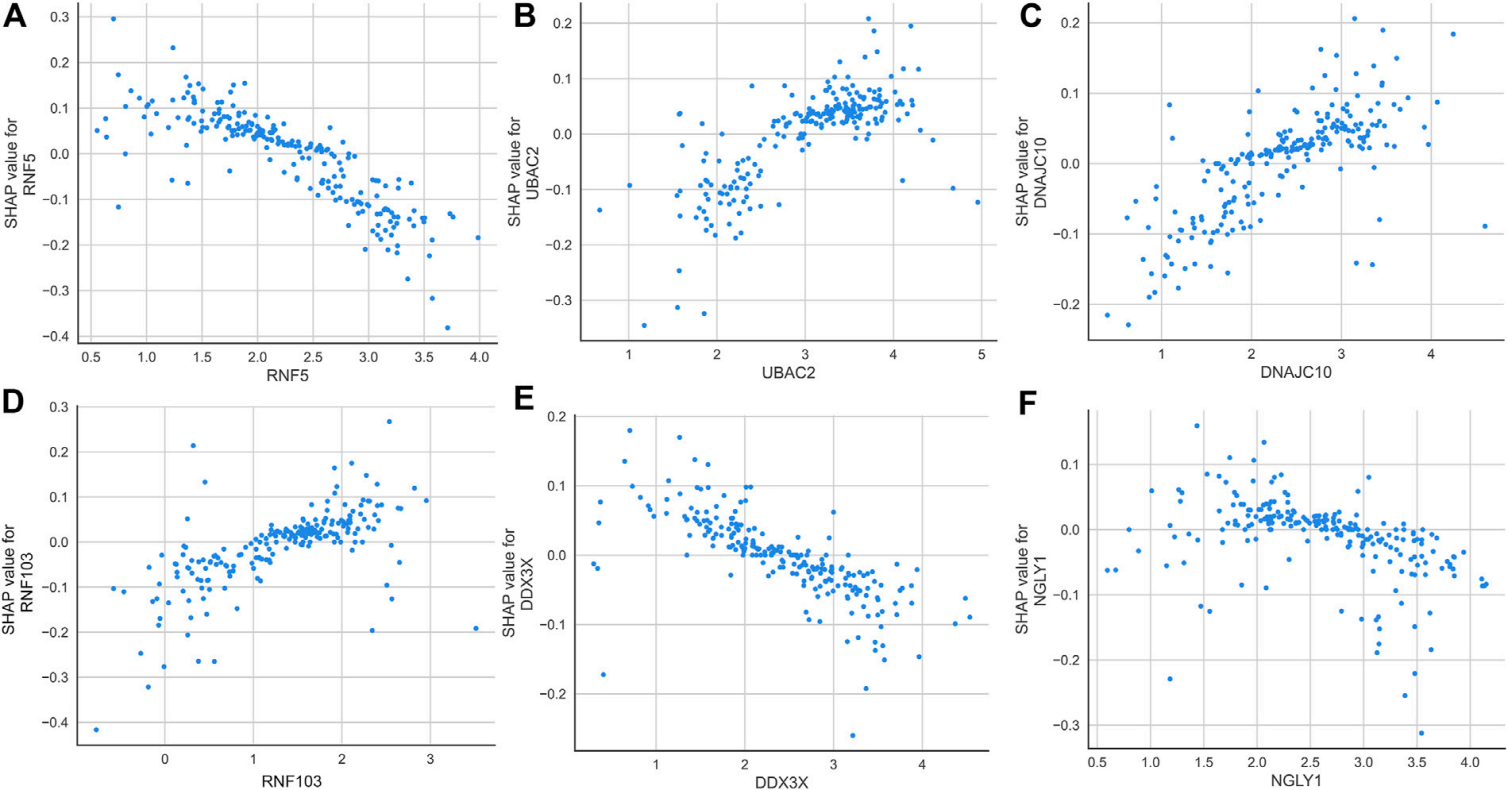
SHAP dependence plot of the SVM model, exhibiting the effect of RNF5 **(A)**, UBAC2 **(B)**, DNAJC10 **(C)**, RNF103 **(D)**, DDX3X **(E)**, and NGLY1 **(F)** on the prediction. SHAP values that exceed zero indicate an increased risk of AD.

According to the average SHAP values, we intersected the top nine ER stress-related DEGs from the SVM and LightGBM machine learning models to determine the final characteristic genes. Following intersection, six characteristic genes shared by SVM and LightGBM algorithms were eventually determined (RNF5, UBAC2, DNAJC10, RNF103, DDX3X, and NGLY1). SHAP dependence plots presented how a characteristic gene influenced the occurrence of AD and exhibited how the attributed importance of a feature gene changed with its value. In total, low expression of RNF5, DD3X, and NGLY1 had an increased risk of developing AD. In addition, high expression of UBAC2, DNAJC10, and RNF103 were the risk factors for AD progression (Figure 7).

**FIGURE 8:**
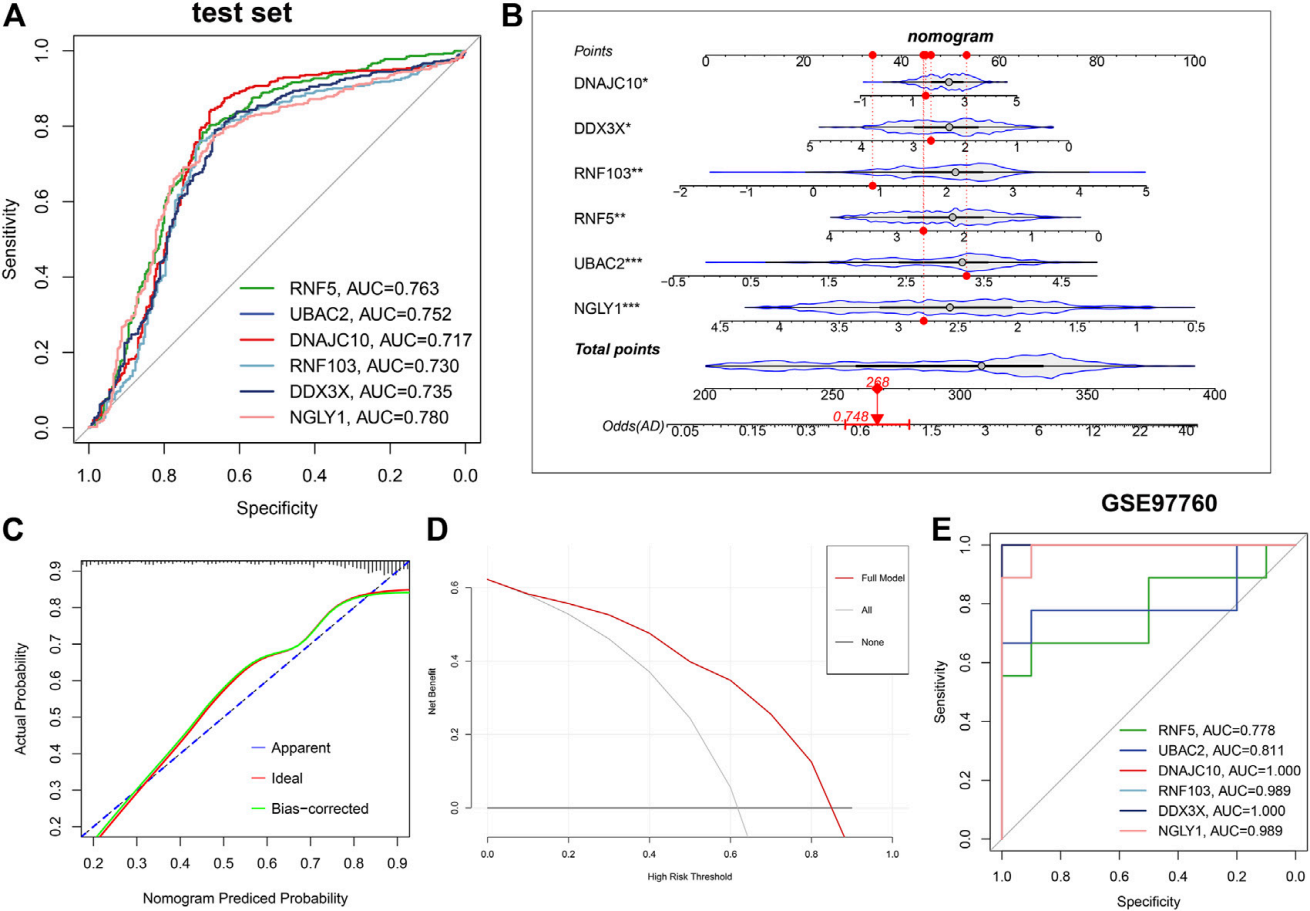
Validation of the diagnostic efficacy based on characteristic genes. **(A)** ROC curves evaluating the diagnostic performance of feature genes in the testing set. **(B)** Establishment of a nomogram for predicting the risk of AD on the basis of feature genes in the testing set. **(C)** Calibration curve evaluates the prediction efficacy of the nomogram. **(D)** DCA estimates the clinical benefit of the nomogram. **(E)** ROC analysis of the six characteristic genes in GSE97760 dataset.

Subsequently, ROC curves analysis was utilized to estimate the diagnostic ability of each feature gene in predicting AD progression in the testing set. The AUC values of ROC curves were 0.763 of RNF5, 0.752 of UBAC2, 0.737 of DNAJC10, 0.730 of RNF103, 0.735 of DDX3X, and 0.780 of NGLY1 (Figure 8A). In addition, a nomogram was established as a predictive tool for AD progression by incorporating these six ER stress-related feature genes. In the nomogram, the expression of each feature variable corresponded to a score point, and the total score corresponded to different AD risks, which was obtained by adding up the scores of all feature variables (Figure 8B). The calibration curve confirmed that the nomogram had the ability to accurately evaluate the progression of AD (Figure 8C). The decision curve analysis indicated that the patients with AD could derive clinically benefit from the nomogram (Figure 8D). Moreover, external validation dataset GSE97760 was further applied for verifying the diagnostic value of these characteristic genes. Consistently, The AUC values of ROC curves were 0.778 of RNF5, 0.811 of UBAC2, one of DNAJC10, 0.989 of RNF103, one of DDX3X, and 0.989 of NGLY1 (Figure 8E). Combined these results, we can infer that these ER stress-related feature genes enabled to accurately distinguish AD from non-AD normal.

### Identification of ER stress subtypes in AD

**FIGURE 9:**
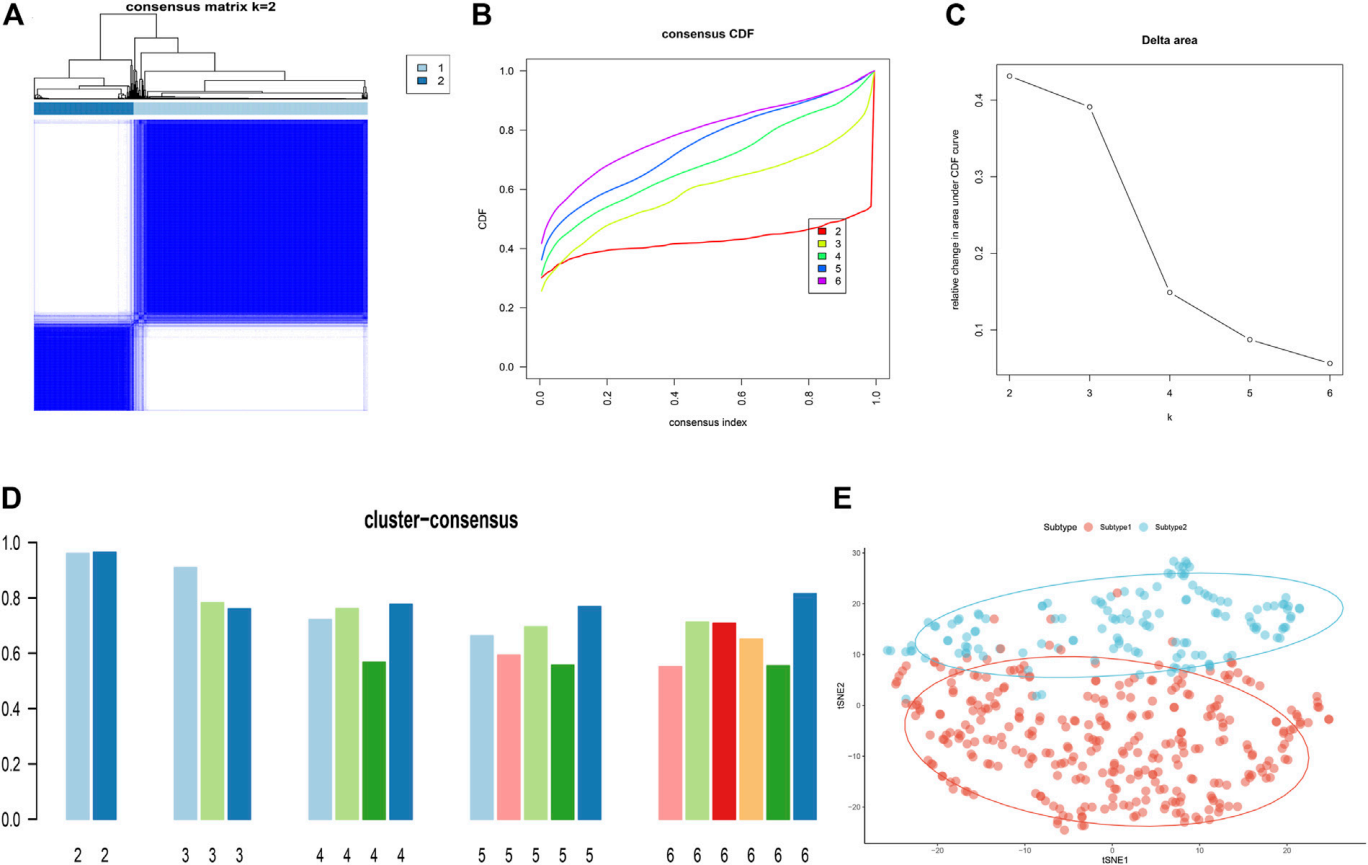
Identification of ER stress-related molecular patterns in AD. **(A)** Consensus clustering matrix when k = 2. **(B–E)** Representative CDF curves when *k* = 2 to 6. **(C)** Relative alterations in CDF delta area curves. **(D)** Consensus score in each subtype when *k* = 2 to 6. **(E)** t-SNE diagram demonstrates that the AD patients are classified into two distinct subtypes.

To illustrate the ER stress-related patterns in AD, we classified the 453 AD samples based on the expression landscapes of seven ER stress-related feature genes using a consensus clustering approach. The number of subtypes were more stable when *k* = 2 (Figure 9A), and the CDF plot exhibited the minimum fluctuation when the consistency index ranged from 0.2 to 0.6 (Figure 9B). Moreover, relative alterations in the area under the CDF curve presented the significant difference (k and k-1) when *k* = 2 to *k* = 6 (Figure 9C). Additionally, the consistency score of each subtype was highest (both more than 0.9) when *k* = 2 (Figure 9D). We thus classified 453 AD samples into two distinct subtypes, including Subtype1 (*n* = 318) and Subtype2 (*n* = 135). tSNE analysis demonstrated the obvious difference between these subtypes (Figure 9E).

### Differentiation of ER stress features and immune characteristics between ER stress subtypes

**FIGURE 10:**
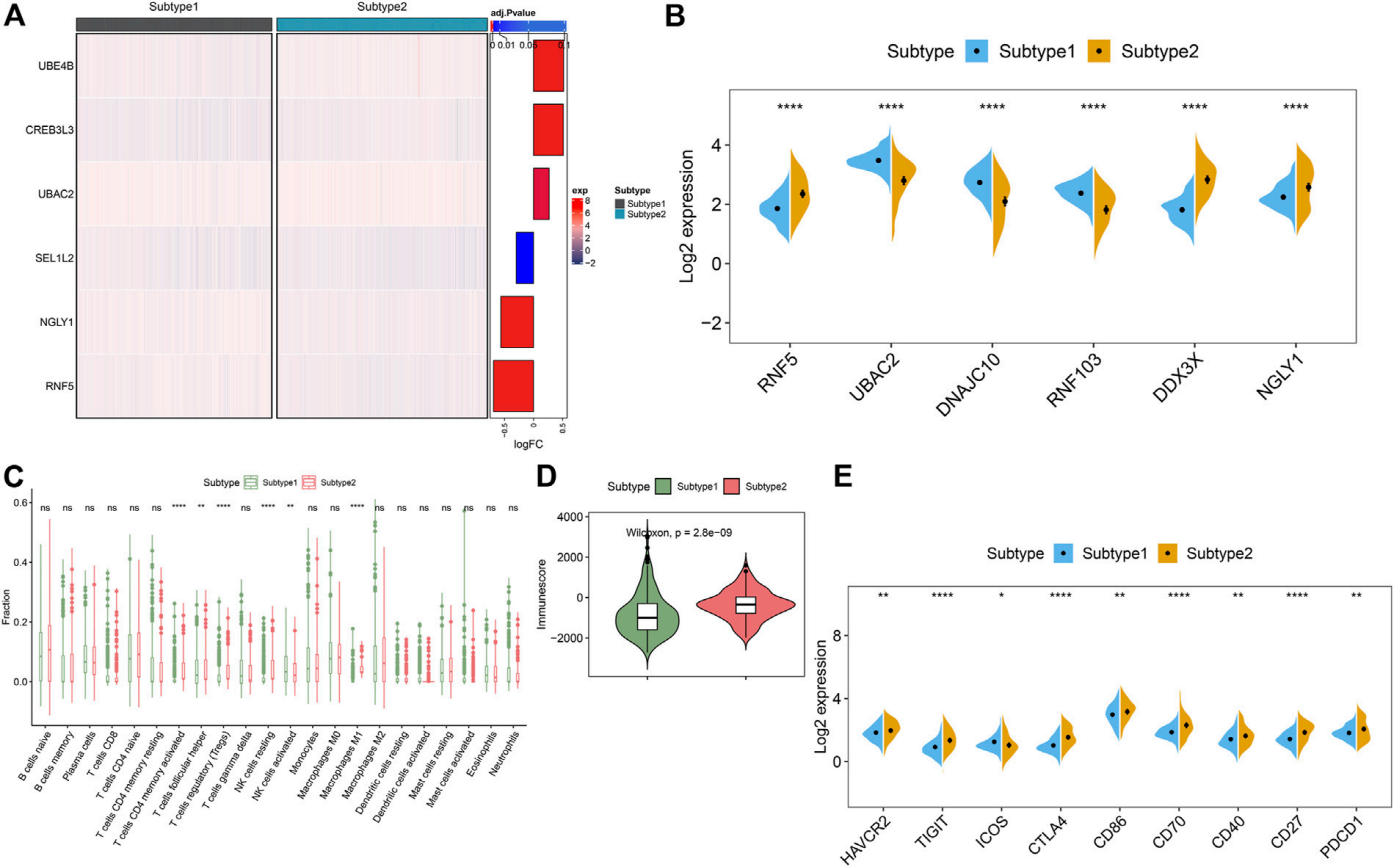
Identification of the differentiation of feature genes and immune characteristics between ER stress subtypes. **(A)** Representative heatmap reveals the differential expression of six characteristic genes between ER stress subtypes. **(B)** Representative boxplots show the expression of six characteristic genes between ER stress subtypes. ****p < 0.0001. **(C)** Representative boxplots show the differences of infiltrated immune cells between ER stress subtypes. **p < 0.01, ****p < 0.0001, ns, no significance. **(D)** Representative boxplot reveals the immune score between ER stress subtypes. **(E)** Representative boxplots present the expression of immune checkpoints-related genes. *p < 0.05. **p < 0.01.****p < 0.0001.

To clarify the molecular characteristics between these subtypes, we first extensively evaluated the expression differences of six ER stress-related feature genes in the groups with different ER stress molecular patterns. The distinct expression profiles of ER stress-related feature gene were found between these ER stress molecular patterns (Figure 10A). Subtype1 presented enhanced expression of UBAC2, DNAJC10, and RNF103, whereas subtype2 was characterized by higher expression of RNF5, DDX3X, and NGLY1 (Figure 10B). Additionally, alternations in immune infiltrating were observed between subtype1 and subtype2. Subtype2 showed greater abundances of follicular helper T cells, regulatory T cells (Tregs), resting NK cells, and M1 macrophages (Figure 10C). Consistently, subtype2 also displayed a higher immune score (Figure 10D). Next, we further estimated the difference of classic immune and immune checkpoint-related genes between these subtypes. The results revealed that most immunosuppression, immune activation, and MHC-related genes were conspicuous boosted in subtype2 when compared with subtype1 (Supplementary Figures S1A–C), indicating that ER stress subtype2 had a stronger immune response than ER stress subtype1. Furthermore, the expression of immune checkpoint-related genes, such as HAVCR2, TIGIT, CTLA4, CD86, CD70, CD40, CD27, and PDCD1, were remarkably upregulated in the ER stress subtype2. Combined these results, we considered ER stress subtype2 as an immune subtype and might be benefit from immune therapy (Figure 10E).

### Identification of functional annotation based on ER stress subtypes

**FIGURE 11:**
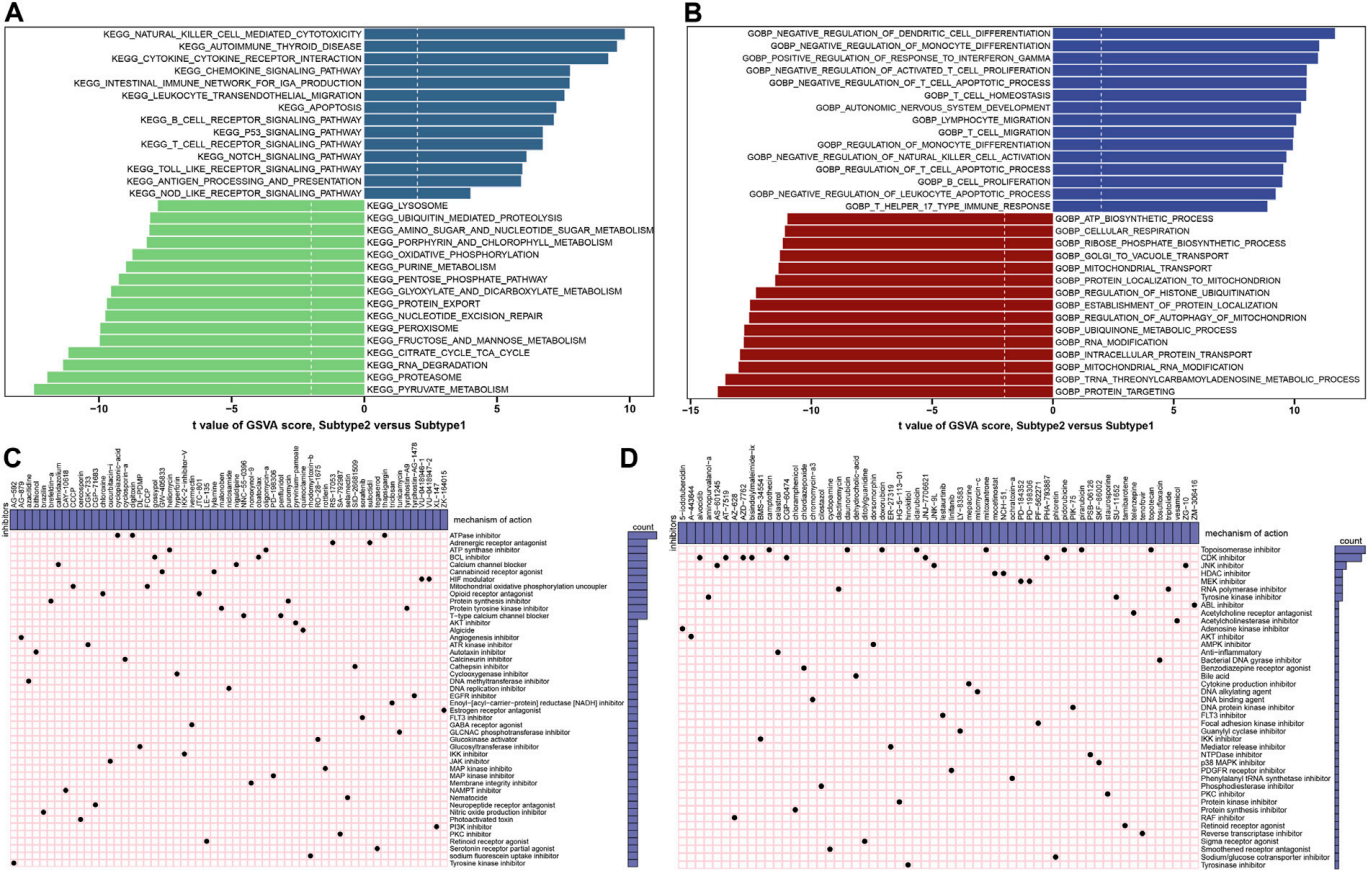
Identification of cluster-specific biological characteristic and small-molecule compounds. **(A)** Differences in the enriched hallmark pathways between ER stress subtypes ranked by t value of GSVA score. **(B)** Differences in enriched biological functions between ER stress subtypes ranked by t value of GSVA score. **(C,D)** CMap analysis shows the MoA based on subtype1-specific **(C)** and subtype2-specific **(D)** small-molecule compounds.

The GSVA analysis was conduct to evaluate the differences of enriched functions and pathways in the groups with different ER stress expression patterns. The results revealed that apoptosis and cytokine associated pathways were upregulated in suptype2. Otherwise, the obvious pathways were also enriched in the regulation of immune responses such as autoimmune thyroid disease, the production of IGA, B cells and T cells receptor signaling pathways, toll like and nod like receptor signaling pathways, and antigen processing and presentation (Figure 11A; Supplementary Table S3). In addition, functional enrichment results suggested that dendritic cells differentiation, the development of nervous system, the regulation of immune-related biological functions such as monocyte differentiation, T cells proliferation and migration, the activation of natural killer cells, lymphocyte migration, T cells and leukocyte mediated apoptotic process were prominently upregulated in subtype2 (Figure 11B; Supplementary Table S4).

### Prediction of subtype-specific small molecular compounds and mechanism of action

To explore the potential drug targets againts distinct subtypes, the prediction of subtype1 and subtype2-sepcific small molecular compounds was performed using CMap analysis. Among the subtype1-specific small molecular compounds, cyclopiazonic-acid, digitoxin, and thapsigargin shared ATPase inhibitors, while gossypol and obatoclax shared BCL inhibitor (Figure 11C). Among the subtype2-specific small molecular compounds, camptothecin, daunorubicin, doxorubicin, idarubicin, mitoxantrone, pidorubicine, pirarubicin, topotecan shared Topoisomerase inhibitor (Figure 11D).

## Discussion

AD, the most prevalent neurodegenerative disease worldwide, can lead to progressive decline of cognitive function, eventually result in death (Kumar et al., 2015). However, due to the poor of the existing neural markers and the heterogeneity of pathogenesis in patients with AD, a great number of patients have not achieved satisfactory results (Mann et al., 1988; Maciejczyk et al., 2020; Cano et al., 2021; Duara and Barker, 2022). Therefore, it is urgent to identify more powerful diagnostic markers and more suitable molecular subtypes and to establish a diagnostic model for AD.

As one of the most critical organelles, Endoplasmic reticulum (ER) mainly participates in the protein synthesis, lipid metabolism, and the storage of intracellular calcium, as well as the regulation of various cellular signaling pathways (Schwarz and Blower, 2016; Kepp and Galluzzi, 2020). The accumulation of misfolded proteins in the ER results in ER stress, eventually activates the protective unfolded protein response (UPR) to maintain the homeostasis of ER (Ron and Walter, 2007). The activation of ER stress-related sensors, such as PERK-ATF4-CHOP, IRE1-XBP1, and pathways exert a vital role in regulating cellular physiological responses induced by ER stress. (Lee, 2005; Yang et al., 2013; Yatchenko et al., 2019). However, excessive and persistent ER stress could lead to inappropriate activation of UPR, eventually resulting in apoptosis or autophagy-dependent cell death (Szegezdi et al., 2006; Fernández et al., 2015). Previous study demonstrated that ER stress is closely related with the occurrence and progression of various diseases including neurodegenerative diseases, and tumors (Hetz and Saxena, 2017; Chen and Cubillos-Ruiz, 2021; Ren et al., 2021). Therefore, the comprehensive exploration of ER stress will provide critical insights into the clinical treatment of these diseases.

Excessive accumulation of misfolded proteins caused by alternations in the expression of ER chaperones and folding protein enzymes is closely related to AD. ER stress characterized by the dysfunction of unfolded protein response could eventually result in neurodegeneration and neuronal death (Gerakis and Hetz, 2018; Uddin et al., 2021). ER stress is usually accompanied by the activation of PERK-dependent phosphorylation signal pathway, which is evidenced by the enhanced downstream expression of ATF4 and CHOP proteins. Subsequently, the pro-apoptotic branch of UPR is activated, eventually triggers neuronal loss and AD progression (Rozpędek et al., 2019). In addition, ER stress plays a critical role in activating immunosuppressive network in AD patients. For example, the myeloid-derived suppressor cells (MDSC) and regulatory T cells (Treg) have been implicated in the progression of AD by exhibiting their repressive phenotypes (Salminen et al., 2020). Besides, relevant studies also demonstrated that the suppression of IRE-1-XBP1 signaling pathway in the nervous system can exert prominent neuroprotective effect by constraining the accumulation of amyloid and amyloid β oligomers, and inhibiting the activation of astrocyte (Duran-Aniotz et al., 2017; Zarini-Gakiye et al., 2021). Although increasing number of studies have confirmed the pathological mechanisms of ER stress in AD, the specific biological functions of ER stress in AD have not been fully elucidated, especially ER stress-related molecular patterns and the roles of ER stress in regulation AD immunity.

In this study, we fully evaluated the expression of ER stress regulators in brain tissues between normal and AD individuals. A total of 17 dysregulated ER stress-related DEGs were determined in AD brain tissues, suggesting a crucial role of ER stress in exacerbating the progression of AD. Correlation analysis revealed the significant synergistic or antagonistic effects among these ER stress regulators, as evidenced by the interactions of ER stress-related DEGs in AD patients. Meanwhile, a significant alternation in the proportion of immune cells was found between normal and AD subjects. AD patients presented greater infiltration levels of CD8^+^ T cells, regulatory T cells (Tregs), gamma delta T cells, Monocytes, M1 macrophages, resting dendritic cells, activated dendritic cells, activated mast cells, eosinophils and neutrophils, which were consensus with the previous studies (Ciaramella et al., 2010; Kwak et al., 2014; Lehrer and Rheinstein, 2016; Stock et al., 2018; Kapellos et al., 2019; Salminen et al., 2020). Furthermore, we also found an extraordinary correlation between these upregulated immune cells and ER stress-related DEGs. Subsequently, we compared the performance of nine machine learning algorithms and the SVM model exhibited the highest AUC value and relative high P-R curve area. Subsequently, we performed the global and local explanation of these ER stress-related genes and selected six characteristic genes (RNF5, UBAC2, DNAJC10, RNF103, DDX3X, and NGLY1), all of them enable to precisely predict AD progression. Further, we performed unsupervised cluster approach to estimate the molecular patterns in AD brain tissues based on the expression six characteristic ER stress regulators, and we eventually identified two distinct molecular subtypes. Immune infiltration analysis indicated that subtype2 displayed a enhanced immune score and relatively greater immune infiltration levels. Functional enrichment analysis revealed that subtype2 was closely related to differentiation, migration, and activation of multiple immune cells. Moreover, immune checkpoint-related genes were remarkably increased in the subtype2. Therefore, it would be reasonable to infer that subtype2 may be able to prevent AD deterioration by generating multiple immune cells such as T cells and B cells, and eventually exhibit a better prognosis for AD.

In recent years, machine learning has been widely used to predict novel biomarkers and provide novel insights into the mechanism of disease pathogenesis due to the excellent performance in clinical diagnosis (Rajkomar et al., 2019; Do and Le, 2020; Le et al., 2021). This study identified potential ER stress-related DEGs for predicting the progression of AD and constructed a predictive model in AD patients on the basis of machine learning models. The predictive performance generated by nine machine learning classifiers were compared based on the expression landscapes of differentially expressed ER stress-related genes, and the results indicated that SVM model had the highest AUC (0.879), accuracy (0.808), true positive predictive (TP = 115), true negative predictive values (TN = 61), recall (0.873), and precision (0.809). It has been reported that SVM model is a high-performance linear-decision surface algorithm that has been applied for the early prediction of AD (Liu et al., 2021; Zhang et al., 2021). To the best of our knowledge, this is the first published study to identify potential biomarkers associated with ER stress through comparing multiple machine learning algorithms and apply the SVM algorithm to predict the risk of AD.

Previous studies have described machine learning algorithms as black boxes because they provide little information about how predictions have been made, which greatly limits the clinical application of machine learning, as clinicians are reluctant to apply opaque decision-making methods for medical diagnosis. In our current study, we utilized SHAP methodology to further explain the decision process of the SVM and LightGBM algorithms, respectively. Subsequently, we identified the final six feature variables (RNF5, UBAC2, DNAJC10, RNF103, DDX3X, and NGLY1) via intersecting the results of SVM and LightGBM machine learning models, all of which could accurately predict the progression of AD. RNF5, a member of E3 ubiquitin protein ligase, exerts the neuroprotection effect as a downstream molecular of the DJ-1/Akt signaling pathway (Zhang et al., 2012). UBAC2, which serves as a regulator of ER-associated protein degradation, plays a critical role in promoting the occurrence and development of malignant tumors (Gu et al., 2020). It has been reported that the mutation of DNAJC protein family may contribute to Parkinson’s disease pathogenesis, suggesting that they may be considered as effective therapeutic targets in neurodegenerative diseases (Roosen et al., 2019). As a vital ERAD (ER-associated degradation)-related E3 ligase, RNF103 is mainly participated in the cytosolic protein homeostasis (Kadowaki et al., 2018). DDX3X is a gene essentially for cortical development by regulating neuron outgrowth. DDX3X mutations can impair RNA helicase activity and disturb RNA metabolism, thus leading to the pathogenesis of autism, brain malformations, and epilepsy (Lennox et al., 2020). Recent study has demonstrated that depletion of NGLY1 is implicated in the presentation of neurodegenerative phenotypes and the development of pathological abnormalities, which might be associated with the accumulation of cytoplasmic ubiquitinated proteins (Asahina et al., 2020).

Some limitations needed to be pointed out in the current study. Firstly, more detailed clinical information needed to be taken into account to validate the predictive efficacy of the SVM machine learning. In addition, subsequent experiments are essentially to confirm the expression levels of ER stress-related DEGs as well as the therapeutic effects of subtype-specific small-molecule compounds in alleviating AD. Moreover, larger number of external validation cohorts are required to construct stability in the performance of our diagnostic model.

## Conclusion

Overall, our study revealed a correlation between ER stress and infiltrated immune cells and determined six feature genes associated ER stress (RNF5, UBAC2, DNAJC10, RNF103, DDX3X, and NGLY1) that could accurately predict AD progression based on interpretable machine learning. Moreover, we illustrated the prominent heterogeneity of immunity between AD patients with distinct ER stress subtypes. Our study provides new insights into the role of ER stress in AD heterogeneity and the development of novel targets for immunotherapy in patients with AD.

## Data Availability

The datasets presented in this study can be found in online repositories. The names of the repository/repositories and accession number(s) can be found in the article/Supplementary Material.
