# Supplementary material for: Identification of endoplasmic reticulum stress-associated genes and subtypes for prediction of Alzheimer’s disease based on interpretable machine learning
Source: Front Pharmacol. 2022 Aug 19;13:975774. doi: 10.3389/fphar.2022.975774 (PMC9438901; doi:10.3389/fphar.2022.975774)
Supplement: Supplementary file 5 [file Image1.pdf]

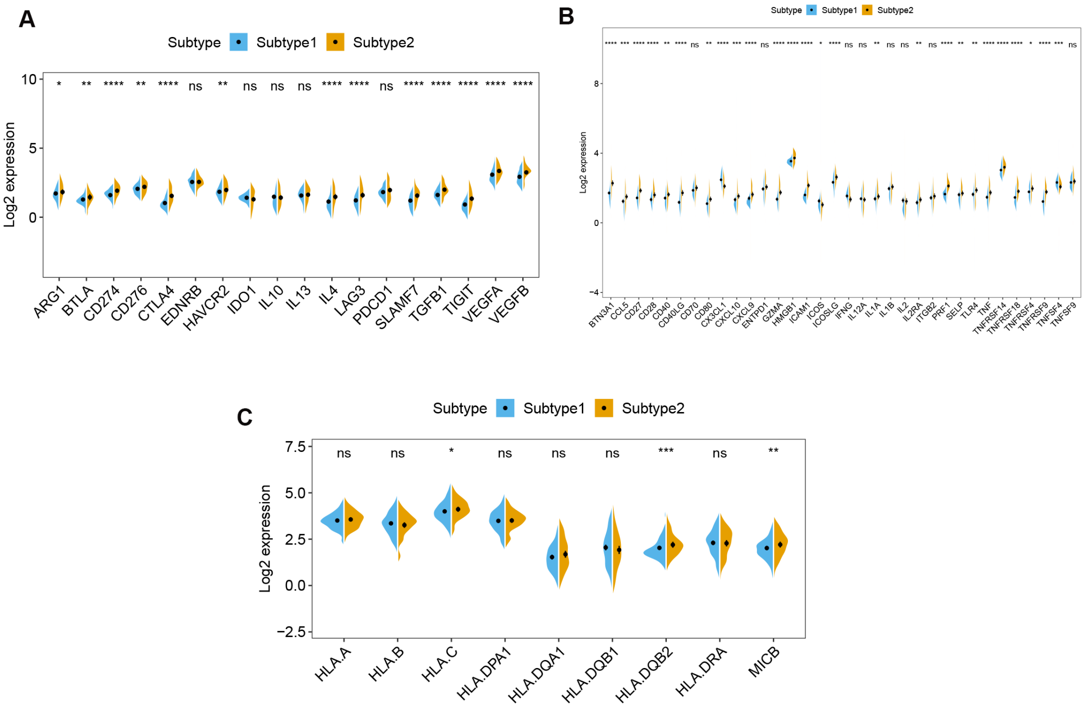

Figure S1 **The expression levels of immunosuppression (A), immune activation (B), and MHC-related genes (C) between subtype1 and subtype2.**
